# Multiple Cross Displacement Amplification Combined with Gold Nanoparticle-Based Lateral Flow Biosensor for Detection of *Vibrio parahaemolyticus*

**DOI:** 10.3389/fmicb.2016.02047

**Published:** 2016-12-22

**Authors:** Yi Wang, Hui Li, Dongxun Li, Kewei Li, Yan Wang, Jianguo Xu, Changyun Ye

**Affiliations:** ^1^State Key Laboratory of Infectious Disease Prevention and Control, National Institute for Communicable Disease Control and Prevention, Collaborative Innovation Center for Diagnosis and Treatment of Infectious Diseases, Chinese Center for Disease Control and PreventionBeijing, China; ^2^Department of Microbiology, Guizhou Medical UniversityGuiyang, China; ^3^Changping District Center for Disease Control and PreventionBeijing, China; ^4^Institute of Microbiology, Jilin Provincial Center for Disease Control and PreventionChangchun, China

**Keywords:** *Vibro parahaemolyticus*, multiple cross displacement amplification, lateral flow biosensor, MCDA-LFB, limit of detection

## Abstract

*Vibrio parahaemolyticus* (*V. parahaemolyticus*) is a marine seafood-borne pathogen causing severe illnesses in humans and aquatic animals. In the present study, multiple cross displacement amplification was combined with a lateral flow biosensor (MCDA-LFB) to detect the *toxR* gene of *V. parahaemolyticus* in DNA extracts from pure cultures and spiked oyster homogenates. Amplification was carried out at a constant temperature (62°C) for only 30 min, and amplification products were directly applied to the biosensor. The entire process, including oyster homogenate processing (30 min), isothermal amplification (30 min) and results indicating (∼2 min), could be completed within 65 min. Amplification product was detectable from as little as 10 fg of pure *V. parahaemolyticus* DNA and from approximately 4.2 × 10^2^ CFU in 1 mL of oyster homogenate. No cross-reaction with other *Vibrio* species and with non-*Vibrio* species was observed. Therefore, the MCDA-LFB method established in the current report is suitable for the rapid screening of *V. parahaemolyticus* in clinical, food, and environmental samples.

## Introduction

*Vibrio parahaemolyticus* (*V. parahaemolyticus*) is a Gram-negative halophilic bacterium that is widely distributed in marine, estuarine, and coastal environments (Letchumanan et al., 2014). The organism is a major food-borne pathogen and frequently isolated from a variety of raw seafoods, such as shellfish, oysters, shrimp, crab, fish and lobster (Wang R. et al., 2015; Letchumanan et al., 2016). In humans, *V. parahaemolyticus* is able to cause acute gastroenteritis after the consumption of raw, undercooked or mishandled seafood (Su and Liu, 2007). The typical clinical symptoms of *V. parahaemolyticus* infection are abdominal pain and acute dysentery, accompanied by fever, chills, headache, nausea, vomiting, diarrhea, and water-like stools (Shimohata et al., 2010). In rare cases, the bacterium is responsible for ear infection, wound infection, or septicaemia that may be life-threatening to populations belonging to special at-risk groups, such as people with immune disorders or liver disease (Centers for Disease Control and Prevention, 2005). In aquatic animals, *V. parahaemolyticus* has the ability to cause serious illnesses in shellfish, fish and penaeid shrimp, resulting in significant losses in aquaculture industries (Tran et al., 2013).

Two hemolysins, thermostable direct hemolysin (TDH) and TDH-related hemolysin (TRH), are major virulence factors for *V. parahaemolyticus* (Letchumanan et al., 2015). Most *V. parahaemolyticus* strains in seafood samples and environment do not harbor these two hemolysin genes, but virulent strains are often found within larger populations of avirulent strains (Su and Liu, 2007; Velazquez-Roman et al., 2012). Since avirulent and virulent isolates have similar growth characteristics, it is difficult to distinguish them phenotypically and therefore, the presence of *V. parahaemolyticus* in general has been as an indicator of seafood contamination.

Traditionally, culture and biochemical methods for identification and detection of *V. parahaemolyticus* from seafood samples can take more than 3 days to complete (Cai et al., 2006). Although PCR-based assays offer more rapid detection, they require instrumentation that might not be readily available in many settings (Levin, 2006; Letchumanan et al., 2014; Law et al., 2015). Multiple cross displacement amplification (MCDA), a novel nucleic acid amplification technique, has been applied in detecting many bacterial agents (Wang Y. et al., 2015; Wang et al., 2016b,c,d). MCDA assay was conducted under isothermal conditions (60–65°C), thus a simple heater or water bath that maintained a uniform temperature was sufficient. MCDA methods are simple, rapid, highly specific and sensitive, and yield amplifcons from as few as three bacterial cells. Detection of these amplicons can be achieved with disposable lateral flow biosensors (LFB; Wang et al., 2016b,d).

In the current report, a MCDA-LFB assay was established for the rapid detection of *V. parahaemolyticus* strains carrying *toxR* gene (*V. parahaemolyticus*-specific gene; Kim et al., 1999). The analytical sensitivity and specificity were determined in pure cultures and in spiked oyster samples.

## Materials and Methods

### Reagents and Instruments

Rabbit anti-fluorescein antibody (anti-FITC Ab) and biotinylated bovine serum albumin (biotin-BSA) were purchased from the Abcam Co., Ltd. (Shanghai, China). Streptavidin-immobilized 30-nm gold nanoparticles (SA-G) was purchased from the Resenbio Co., Ltd. (XiAn, China). Membrane backing materials, sample and conjugate pads, nitrocellulose membrane (NC), and absorbent pads were purchased from the Jie Yi Biotechnology Co., Ltd. (Shanghai, China). The Loopamp kits were purchased from Eiken Chemical Co., Ltd. (Beijing, China). QIAamp DNA Mini Kit (QIAamp DNA minikits; Qiagen, Hilden, Germany) was purchased from Qiagen Co., Ltd. (Beijing, China). The visual detection reagent (Hydroxynaphthol blue, HNB) was purchased from BeiJing-HaiTaiZhengYuan Technology Co., Ltd. (Beijing, China).

### Preparation of Gold Nanoparticle-Based Dipstick Biosensor

The dry-reagent strips (4 mm × 60 mm) were prepared as previously described with some modifications (Wang et al., 2016b,d). In brief, the sample pad, conjugate pad, NC membrane and absorbent pad were laminated onto a plastic adhesive backing card. The anti-FITC Ab (0.15 mg/ml) and biotin-BSA (2.5 mg/ml) conjugates were sprayed onto NC membrane to form the test line (TL) and control line (CL), with each line separated by 5 mm. SA-G in 0.01M PBS (PH 7.4) was deposited on the conjugate pad of the strip. The assembled cards were cut into 4-mm wide strips (Deli No. 8012). The assembled biosensors were packaged in a plastic box containing a desiccant gel and stored at the room temperature.

### Bacterial Strains and Genomic Template Preparation

The strains employed in this study (**Table 1**) were stored in 10% (w/v) glycerol broth at -70°C. The *Vibrio* isolates were cultured three times on thiosulfate citrate bile salt sucrose agar (TCBS agar, Eiken Chemical) at 35°C and the non-*Vibrio* strains were cultured three times on nutrient agar plate at 37°C. Genomic DNA was extracted from all culture strains using the QIAamp DNA Mini Kit according to the manufacturer’s instructions and quantified using a Nano drop ND-1000 instrument (Calibre, Beijing, China). *V. parahaemolyticus* ICDC-NVP001 was serially diluted (10 ng, 10 pg, 10 fg, 1 fg, and 0.1 fg) for sensitivity analysis of *V. parahaemolyticus*-MCDA-LFB detection.

**Table 1:** Bacterial strains used in this study.

Bacteria	Strain no. (source of strains) ^a^	No. of strains
*Vibrio parahaemolyticus*	ICDC-NVP001	1
	Isolated strains	99
*Vibrio vulnificus*	ATCC27562	1
	Isolated strains	4
*Vibrio cholerae*	ATCC14035	1
	Isolated strains	4
*Vibrio mimicus*	Isolated strains	1
*Vibrio fluvialis*	Isolated strains	1
*Vibrio alginolyticus*	Isolated strains	1
*Plesiomonas shigelloides*	Isolated strains	1
*Aeromonas hydrophila*	Isolated strains	1
*Enterohemorrhagic E. coli*	EDL933	1
*Enteropathogenic E. coli*	Isolated strains	1
*Enterotoxigenic E. coli*	Isolated strains	1
*Enteroaggregative E. coli*	Isolated strains	1
*Enteroinvasive E. coli*	Isolated strains	1
*Shigella dysenteriae*	Isolated strains	1
*Shigella boydii*	Isolated strains	1
*Shigella flexneri*	Isolated strains	1
*Shigella sonnei*	Isolated strains	1
*Salmonella*	Isolated strains	1
*Enterococcus faecalis*	ATCC35667	1
*Enterococcus faecium*	Isolated strains	1
*Listeria monocytogenes*	EGD-e	1
*Listeria ivanovii*	ATCCBAA-678	1
*Listeria grayi*	ATCC25402	1
*Listeria innocua*	Isolated strains	1
*Listeria welshimeri*	Isolated strains	1
*Listeria seeligeri*	Isolated strains	1
*Yersinia enterocolitica*	ATCC23715	1
*Enterobacter cloacae*	Isolated strains	1
*Bntorobater sakazakii*	Isolated strains	1
*Bacillus cereus*	Isolated strains	1
*Campylobacter jejuni*	ATCC33291	1
*Pseudomonas aeruginosa*	Isolated strains	1
*Staphylococcus aureus*	Isolated strains	1
*Staphylococcus epidermidis*	Isolated strains	1
*Staphylococcus saprophyticus*	Isolated strains	1
*Klebsiella pneumoniae*	Isolated strains	1


*^a^ATCC, American Type Culture Collection; ICDC, National Institute for Communicable Disease Control Disease Control and Prevention, Chinese Center for Disease Control and Prevention.*

### Design of MCDA Assay Primers

**FIGURE 1:**
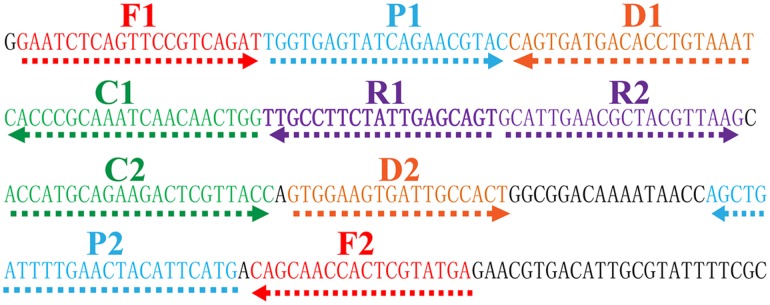
**Location and sequence of *toxR* gene (*Vibrio parahaemolyticus*-specific gene) used to design multiple cross displacement amplification primers.** The nucleotide sequence of the sense strand of *toxR* gene was shown. Right arrows and left arrows indicate sense and complementary sequences that were used.

The MCDA primer pairs (F1, F2, CP1, CP2, C1, C2, D1, D2, R1 and R2) were designed using PrimerExplorer V4 (Eiken Chemical, Japan) and primer software PRIMER PREMIER 5.0. All primers were analyzed for hairpin structures and hybrids using the Integrated DNA Technologies design tools^1^. Blast analysis was used to verify that the MCDA primers were specific for *V. parahaemolyticus*. The CP1 (C1+P1) and C1 primers were labeled at their 5′ end with biotin and fluorescein isothiocyanate (FITC), respectively. The sequences, positions and modifications of the primer pairs are displayed in **Figure 1** and **Table 2**. All of the oligomers were synthesized and purified by TsingKe Biotech Co., Ltd. (Beijing, China) at HPLC purification grade.

**Table 2:** The primers used in this study.

Primers^a^	Sequences and modifications (5′-3′)	Length^b^	Gene
F1	GAATCTCAGTTCCGTCAGAT	20 nt	*toxR*
CP1	CCAGTTGTTGATTTGCGGGTGTGGTGAGTATCAGAACGTAC	41 mer	
CP1^∗^	Biotin-CCAGTTGTTGATTTGCGGGTGTGGTGAGTATCAGAACGTAC	41 mer	
C1	CCAGTTGTTGATTTGCGGGTG	21 nt	
C1^∗^	FITC-CCAGTTGTTGATTTGCGGGTG	21 nt	
D1	ATTTACAGGTGTCATCACTG	20 nt	
R1	ACTGCTCAATAGAAGGCAA	19 nt	
R2	GCATTGAACGCTACGTTAAG	20 nt	
D2	GTGGAAGTGATTGCCACT	18 nt	
C2	ACCATGCAGAAGACTCGTTACC	22 nt	
CP2	ACCATGCAGAAGACTCGTTACCCATGAATGTAGTTCAAAATCAGCT	46 mer	
F2	TCATACGAGTGGTTGCTG	18 nt	


*^a^CP1^∗^, 5**′**-labeled with biotin when used in MCDA-LFB assay; C1^∗^, 5**′**-labeled with FITC when used in MCDA-LFB assay. ^b^nt, nucleotide; mer, monomeric.*

### The Standard MCDA Assay

Multiple cross displacement amplification reactions were carried out in 25 μl amplification mixtures as previous studies (Wang Y. et al., 2015; Wang et al., 2016b,c,d). Briefly, each reaction contained 0.4 μM each of displacement primers F1 and F2, 0.8 μM each of amplification primers C1^∗^ and C2, 1.2 μM each of amplification primers R1, R2, D1 and D2, 1.2 μM each of cross primers CP1 and CP1^∗^, 2.4 μM cross primer CP2, 12.5 μl 2× reaction mix (Loopamp DNA amplification Kit), 1.25 μl of *Bst* DNA polymerase (10 U) and 1 μl DNA template.

A total of four monitoring methods, including colorimetric indicator (HNB), gel electrophoresis, turbidimeter (LA-320C) and LFB detection, were used for analyzing the amplicons. When employing HNB, the amplified products caused a color change from violet to sky blue, while the negative controls and blank control remained violet. MCDA products were analyzed by 2% agarose gel electrophoresis, the specific ladder of multiple bands should be seen for positive amplifications, but not in the negative and blank controls. By LFB, two visible red lines (TL; CL) should be observed in positive reactions, and only the control lines were visual in negative and blank controls.

The optimal reaction temperature was determined in the range of 60 to 67°C for 60 min. Mixtures with 1 μl genomic template of *Enterococcus faecalis* strains (*E. faecalis*, ATCC35667) and *Shigella flexneri* (*S. flexneri*, ICDC-NPS001) strains were used as negative controls, and mixtures with 1 μl double distilled water (DW) were selected as a blank control.

### Specificity and Sensitivity of the *V. parahaemolyticus*-MCDA-LFB Assay

The specificity of *V. parahaemolyticus*-MCDA-LFB was analyzed with DNA templates from 143 bacterial strains (**Table 1**). The assays were repeated at least twice. The limit of detection (LoD), which was tested using serial dilutions (10 ng, 10 pg, 10 fg, 1 fg and 0.1 fg per microliter), was defined by genomic DNA amount of the template. Detection by *V. parahaemolyticus*-MCDA-LFB was compared to that with a colorimetric indicator (HNB), real time turbidity and 2% agarose gel electrophoresis. Three replicates of each dilution were tested.

### Optimization the Amplification Time of the *V. parahaemolyticus*-MCDA-LFB Assay

The optimal time for *V. parahaemolyticus*-MCDA-LFB was determined by increasing the reaction time from 10 to 40 min at 10 min intervals. The MCDA products were analyzed using LFB detection and two replicates of each amplification time were determined.

### *V. parahaemolyticus*-MCDA-LFB Detection in Oyster Samples

*Vibrio parahaemolyticus* ICDC-NVP001 was added into oyster samples obtained from local seafood restaurants in Beijing. Only oyster samples that tested negative for *V. parahaemolyticus* according to Kim et al. (1999) were spiked. *V. parahaemolyticus* cultures were serially diluted (10^-1^ to 10^-8^), and 100-μl aliquots (appropriate dilution:10^-6^) were placed in triplicate on brain heart infusion (BHI). CFUs were counted after 24 h at 37°C. Simultaneously, 0.1 mL of diluted *V. parahaemolyticus* cultures (10^-3^ to 10^8^) with known amounts (4.2 × 10^5^ to 4.2 × 10^0^ CFU/mL) was inoculated into 900 μl of oyster homogenates and mixed well. The spiked oyster samples were centrifuged at 200 g for 5 min, and the supernatant was placed into a new tube, and the was centrifuged at 18000 g for 5 min. The supernatant was removed and the pellet was subjected to extract genomic DNA, and the DNA templates were eluted in 20 μl of elution buffer. For the MCDA-LFB assay, 1 μl of the extracted DNA was used as template and non-contaminated oyster samples were served as negative control. Three independent assays were performed.

## Results

### Confirmation and Detection of *V. parahaemolyticus* MCDA Products

**FIGURE 2:**
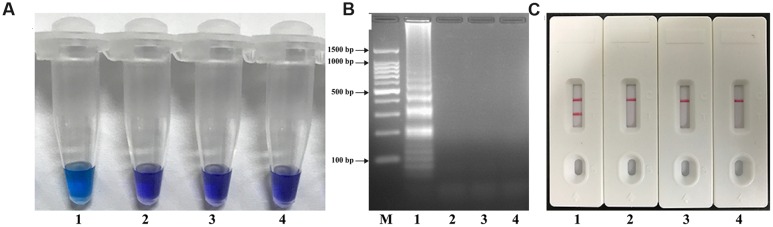
**Confirmation and Detection of *V. parahaemolyticus*-MCDA products.** **(A)** By Hydroxynaphthol blue (HNB), amplification products of *V. parahaemolyticus*-MCDA assay were visually analyzed by observation of the color change. **(B)** Agarose gel electrophoresis of *V. parahaemolyticus*-MCDA products was shown. **(C)** Lateral flow biosensor applied for visual detection of *V. parahaemolyticus-*MCDA products. Tuble 1/Lane 1/Biosensor 1, positive amplification of *V. parahaemolyticus* strain (ICDC-NVP001); Tuble 2/Lane 2/Biosensor 2, negative control of *E. faecalis* strain (ATCC35667); Tuble 3/Lane 3/Biosensor 3, negative control of *S. flexneri* strain (ICDC-NPS001); Tuble 4/Lane 4/Biosensor 4, blank control (DW). Lane M, DNA maker DL 100.

To determine the availability of *V. parahaemolyticus*-MCDA primers (**Table 2**), *V. parahaemolyticus* MCDA assays with DNA from pure cultures were carried out at 62°C for 1 h. Amplification occurred with DNA from *V. parahaemolyticus* (ICDC-NVP001), but not with *E. faecalis* (ATCC 35667), *S. flexneri* (ICDC-NPS001) DNA and the DW control (**Figure 2**). Therefore, the *V. parahaemolyticus*-MCDA primer set was a good candidate for development of the MCDA-LFB assay for *V. parahaemolyticus* detection.

### Optimization of the Temperature for *V. parahaemolyticus*-MCDA-LFB Assay

**FIGURE 3:**
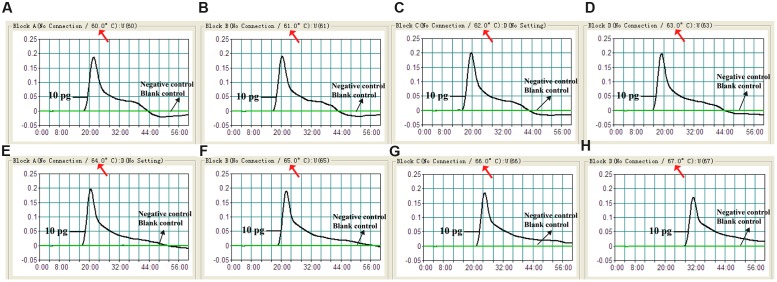
**Optimal reaction temperature for *V. parahaemolyticus*-MCDA primer sets.** The standard MCDA reactions for detection of *V. parahaemolyticus* were monitored by real-time measurement of turbidity and the corresponding curves of concentrations of DNA were marked in the figures. The threshold value was 0.1 and the turbidity of >0.1 was considered to be positive. Eight kinetic graphs **(A–H)** were generated at various temperatures (60–67°C, 1°C intervals) with target pathogens DNA at the level of 10 pg per reaction. The graphs from **B–E** showed robust amplification.

To verify the optimum amplification temperature, *V. parahaemolyticus* strain (ICDC-NPV001) was used as the positive control at the level of 10 pg per reaction and the reactions were monitored by the real time turbidity method. Carrying out MCDA at temperatures from 60 to 67°C at 1°C increments confirmed that 62°C was the most suitable temperature for amplification as indicated by the kinetics graphs displayed in **Figure 3**. At all tested temperatures, the typical kinetics graphs were generated, with the faster amplification obtained from assay temperatures of 62°C.

### Specificity and Sensitivity for *V. parahaemolyticus* Detection by MCDA-LFB

**FIGURE 4:**
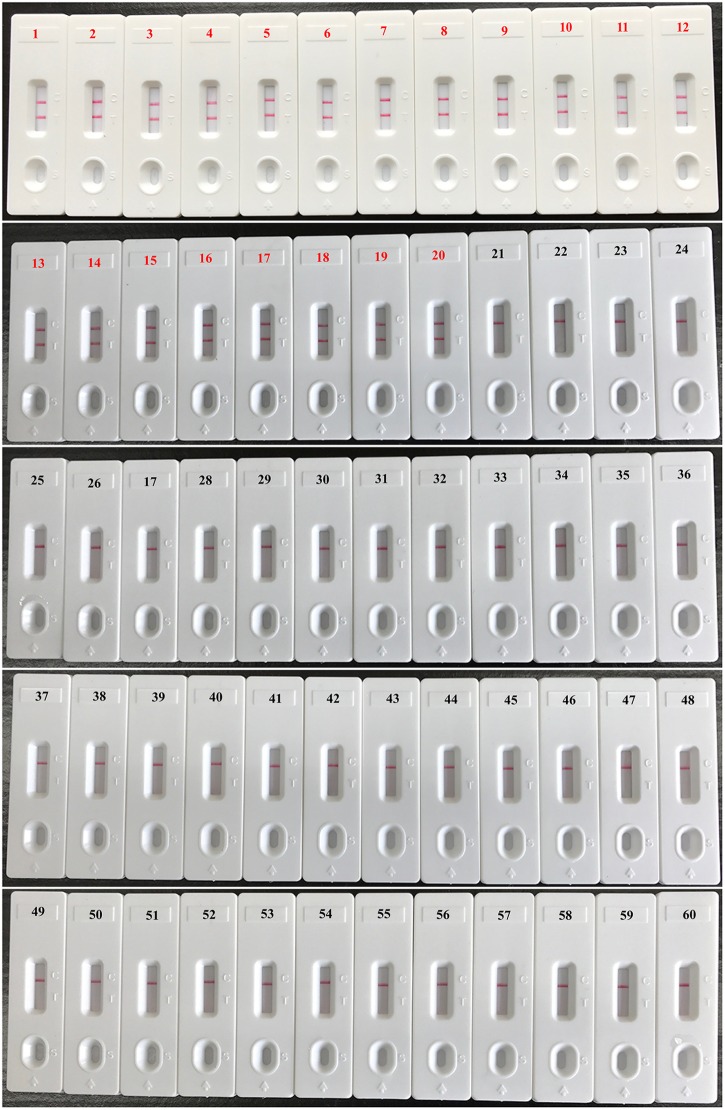
**Analytical specificity of *V. parahaemolyticus*-MCDA-LFB assay for different strains.** The MCDA reactions were carried out using different genomic DNA templates and were analyzed by means of visual format. Biosensor 1, *V. parahaemolyticus* strain (ICDC-NVP001); biosensors 2–20, nineteen isolated strains of *V. parahaemolyticus*; biosensor 21, *V. Cholerae* strain (ATCC14035); biosensors 22–23, two isolated strains of *V. cholerae*; biosensor 24, *V. vulnificus* (ATCC27562); biosensors 25–26, two isolated strains of *V. vulnificus*; biosensor 27 isolated strain of *V. mimicus*; biosensor 28, isolated strain of *V. fluvialis*; biosensor 29, isolated strain of *V. alginolyticus*; biosensors 30–59, *Plesiomonas shigelloides*, *Aeromonas hydrophila*, *Enteropathogenic E. coli*, *Enterotoxigenic E. coli*, *Enteroaggregative E. coli*, *Enteroinvasive E. coli*, *Enterohemorrhagic E. coli*, *Shigella dysenteriae*, *Shigella boydii, Shigella flexneri*, *Shigella sonnei*, *Salmonella*, *Enterococcus faecali*s, *Enterococcus faecium*, *Listeria monocytogenes*, *Listeria ivanovii*, *Listeria grayi*, *Listeria innocua*, *Listeria welshimeri*, *Listeria seeligeri*, *Yersinia enterocolitica*, *Enterobacter cloacae*, *Bntorobater sakazakii*, *Bacillus cereus*, *Campylobacter jejuni*, *Pseudomonas aeruginosa*, *Staphylococcus aureus*, *Staphylococcus epidermidis*, *Staphylococcus saprophyticus*, *Klebsiella pneumoniae*, biosensor 60, blank control (DW).

When DNA from the bacteria listed in table 1 was used in MCDA-LFB assays, only the DNA from the *V. parahaemolyticus* strains provided results. DNA from other *Vibrio* species and from all non-*Vibrio* isolates did not lead to the production of detectable amplification products (**Figure 4**). Two red lines, including TL and CL, appeared on the strips for the positive tests, and only a red line (CL) appeared on the biosensors, suggesting negative results for non-*V. parahaemolyticus* isolates and blank control.

**FIGURE 5:**
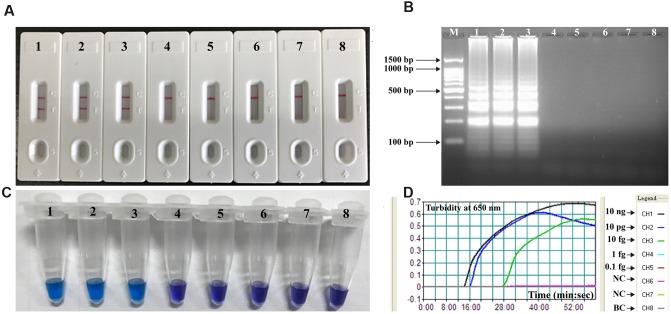
**Analytical sensitivity of MCDA-LFB assay using serially diluted genomic DNA with *V. parahaemolyticus* strain ICDC-NVP001.** A total of four monitoring techniques, including later flow biosensor **(A)**, gel electrophoresis **(B)**, colorimetric indicator (HNB; **C)** and real time turbidity **(D)**, were applied for analyzing the amplification products. The serial dilutions (10 ng, 10 pg, 10 fg, 1 fg, and 0.1 fg) of target templates were subjected to standard MCDA reactions. Biosensors **(A)**/ Lanes **(B)**/Tubes **(C)**/ Turbidity signals **(D)** 1–8 represented the DNA levels of 10 ng, 10 pg, 10 fg, 1 fg and 0.1 fg per reaction, negative control (10 pg of *E. faecalis* genomic DNA), negative control (10 pg of *S. flexneri* genomic DNA) and blank control (DW). The genomic DNA levels of 10 ng, 10 pg and 10 fg per reaction produced the positive reactions.

**FIGURE 6:**
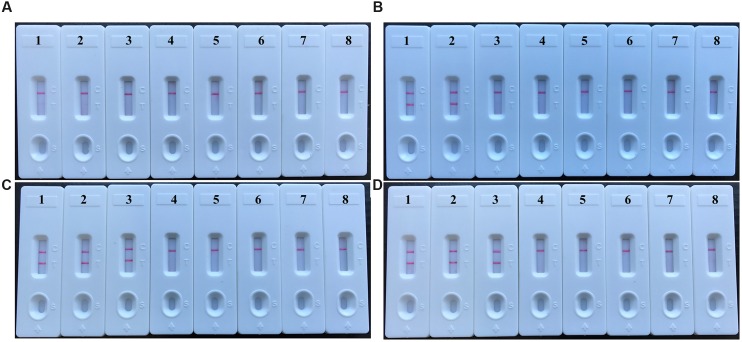
**The optimal duration of time required for *V. parahaemolyticus*-MCDA-LFB assay.** Four different reaction times (**A**, 10 min; **B**, 20 min; **C**, 30 min; **D**, 40 min) were evaluated and compared at 62°C. Biosensors 1, 2, 3, 4, 5, 6, 7, and 8 represent DNA levels of 10 ng of *V. parahaemolyticus* templates, 10 pg of *V. parahaemolyticus* templates, 10 fg of *V. parahaemolyticus* templates, 1 fg of *V. parahaemolyticus* templates, 0.1 fg *V. parahaemolyticus* templates per tube, negative control (*E. faecalis*, 10 pg per reaction), negative control (*S. flexneri*, 10 pg per reaction) and blank control (DW). The best sensitivity was observed when the amplification lasted for 30 min **(C)**.

Serial dilution of the *V. parahaemolyticus* genomic DNA in triplicate and use in MCDA assays demonstrated that as little as 10 fg of template DNA produced sufficient amplified DNA for detection by the four monitoring methods employed (**Figure 5**). The results obtained using biosensor were in complete accordance with real time turbidity, HNB reagent and agarose gel electrophoresis analysis (**Figure 5**). Moreover, it was possible to detect the amplicons when the amplification reaction was carried out for 30 min (**Figure 6**).

### Application of MCDA to *V. parahaemolyticus*-Spiked Oyster Homogenates

**FIGURE 7:**
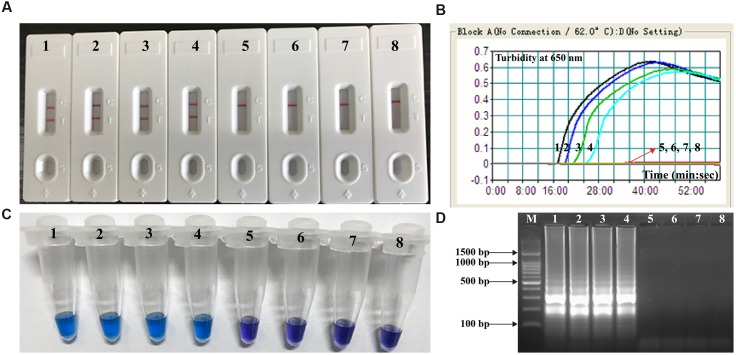
**Analytical sensitivity of *V. parahaemolyticus*-MCDA-LFB for detecting target pathogen in oyster samples.** Four monitoring techniques, including later flow biosensor **(A)**, real time turbidity **(B)**, colorimetric indicator (HNB; **C)** and gel electrophoresis **(D)**, were applied for analyzing the amplification products. The serial dilutions of target templates were subjected to standard MCDA reactions. Strips **(A)**/Turbidity signals **(B)**/ Tubes **(C)**/Lanes **(D)** 1–8 represented the DNA levels of 2100 CFU, 210 CFU, 21 CFU, 2.1 CFU, 0.21 CFU, and 0.021 CFU per reaction, negative control (non-contaminated oyster sample) and blank control (DW). The genomic DNA levels of 2100 CFU, 210 CFU, 21 CFU, and 2.1 CFU per reaction produced the positive reactions.

The lowest number of *V. parahaemolyticus* CFUs could be detected in 1 mL of spiked oyster homogenate was approximately 4.2 × 10^2^ CFU/mL (∼2.1 CFU per reaction; **Figure 7**). The *V. parahaemolyticus*-MCDA assay produced negative results at the concentrations lower than 4.2 × 10^1^ CFU/mL (∼0.21 CFU per reaction), negative control and blank control. As seen with MCDA assays utilizing DNA from pure cultures, detection of the amplicons was as sensitive with the LFB method with the other three methods (**Figure 7**).

## Discussion

The present study demonstrated that multiple cross displacement amplification combined a lateral flow biosensor (MCDA-LFB) utilizing the *toxR* gene as amplification target is capable of detection *V. parahaemolyticus* with excellent specificity and sensitivity. The high level of specificity of the assay is likely due to the utilization of *toxR* as target gene. Although the sequences of housekeeping genes (*rpoD*, *rctB*, *pyrH*, *recA*, and *gyrB*) and 16S rRNA gene have also been considered as possible targets, *toxR* is regarded as gene providing the highest level of discrimination according to phylogenetic tree analysis (Yamamoto and Harayama, 1998; Le Roux et al., 2005; Pascual et al., 2010). Then, the assay’s specificity was successfully examined using pure cultures and oyster samples. The test was positive for all *V. parahaemolyticus* isolates, but negative for other *Vibrio* spp. and non-*Vibrio* isolates (**Figure 4**). Hence, the *V. parahaemolyticus*-MCDA-LFB method provided a high degree of selectivity for identifying *V. parahaemolyticus* strains.

In additional to its sufficient specificity, the newly established *V. parahaemolyticus*-MCDA-LFB method was able to detect as little as 10 fg of *V. parahaemolyticus* DNA isolated from a pure culture (**Figure 5**). The *V. parahaemolyticus-*MCDA-LFB assay was 25-fold more sensitive than *V. parahaemolyticus-*LAMP method, which only detected 250 fg of template DNA per reaction (Wang et al., 2016a). The detection limit of approximately 4.2 × 10^2^ CFU in 1 mL (∼2.1 CFU per reaction) of oyster homogenate was also lower than that of the *V. parahaemolyticus-*LAMP assay (92 CFU per reaction in spiked oyster samples; **Figure 7**) (Wang et al., 2016a). Although the amplification products could be detected equally with other three methods employed in the current study, LFB is likely the preferred method as reading the results is less subjective and does not require instrumentation.

The *V. parahaemolyticus*-MCDA-LFB assay only required a simple incubation at 62°C for 30 min. A variety of portable user-friendly instruments adapted for MCDA reaction exist, the dry block heater (HDT-100C, HengAo, Tianjing, China) being one example. The portable (18 cm × 22 cm), battery-powered device supports 96 MCDA reactions per assay. The MCDA amplification can be conducted using the commercial isothermal amplification kits (such as Eiken Loopamp kits and NEB Warmstart kits), and an MCDA reaction costs approximately $3.5 USD. The cost of LFB is estimated to be $2 USD per test. Combined with the elimination of labor costs because of the requirements for trained personnel in a certified laboratory, our assay becomes more cost-effective.

The MCDA products were directly analyzed using the biosensor (**Figures 2** and **4**–**7**). The entire procedure, including specimen processing (30 min), isothermal reaction (30 min) and detection (1 min), could be finished with 65 min. Detection of amplification products with a lateral flow device is not only fast, but also simpler and less error-prone than detection by the other methods employed in the current study (Zhang et al., 2014).

## Conclusion

A reliable *toxR*-MCDA-LFB assay was successfully established for identification of *V. parahaemolyticus*, causing seafood-borne gastroenteritis in human, which could facilitate investigations to detect the etiological agent of food poisoning, surveillance for *V. parahaemolyticus* contamination in seafood, as well as ecological studies related with regions, practices and environmental factors. The MCDA-LFB approach devised here was sensitive, specific and simple, and did not rely on complicated instrument and expensive reagents. The use of lateral flow biosensor could provide an objective, rapid and easily interpretable readout of the method’s results. Therefore, the *toxR*-MCDA-LFB method could be regarded as a valuable tool for the rapid screening of *V. parahaemolyticus* isolates in clinical, food and environmental samples, especially, in resource-limited areas of developing countries during epidemic periods.

## Author Contributions

YiW, JX, and CY conceived and designed the experiments. YiW, HL, DL, KL, and YaW performed the experiments. YiW, HL, and DL analyzed the data. YiW, HL, DL, KL, and YaW contributed reagents/materials/analysis tools. YiW performed the software. YiW, JX, and CY wrote the paper.

## Disclosures

YW and CY have filed for a patent from the State Intellectual Property Office of the People’s Republic of China, which covers the novel method and sequences included in this manuscript (Application number CN 201611105333.6).

## Conflict of Interest Statement

The authors declare that the research was conducted in the absence of any commercial or financial relationships that could be construed as a potential conflict of interest.

## Footnotes

1 http://www.idtdna.com/pages/scitools
